# Reactivity of Waterlogged Archeological Elm Wood with Organosilicon Compounds Applied as Wood Consolidants: 2D ^1^H–^13^C Solution-State NMR Studies

**DOI:** 10.3390/molecules27113407

**Published:** 2022-05-25

**Authors:** Magdalena Broda, Daniel J. Yelle

**Affiliations:** 1 Department of Wood Science and Thermal Techniques, Faculty of Forestry and Wood Technology, Poznan University of Life Sciences, ul. Wojska Polskiego 38/42, 60-637 Poznan, Poland; 2 Forest Biopolymers Science and Engineering, Forest Products Laboratory, USDA Forest Service, One Gifford Pinchot Drive, Madison, WI 53726, USA; daniel.j.yelle@usda.gov

**Keywords:** archaeological wood, silane, siloxane, wood consolidation, 2D NMR, chemical reactivity, solution-state NMR, wood conservation, waterlogged wood

## Abstract

Some organosilicon compounds, including alkoxysilanes and siloxanes, proved effective in stabilizing the dimensions of waterlogged archaeological wood during drying, which is essential in the conservation process of ancient artifacts. However, it was difficult to determine a strong correlation between the wood stabilizing effect and the properties of organosilicon compounds, such as molecular weight and size, weight percent gain, and the presence of other potentially reactive groups. Therefore, to better understand the mechanism behind the stabilization effectiveness, the reactivity of organosilicons with wood polymers was studied using a 2D ^1^H–^13^C solution-state NMR technique. The results showed an extensive modification of lignin through its demethoxylation and decarbonylation and also the absence of the native cellulose anomeric peak in siloxane-treated wood. The most substantial reactivity between wood polymers and organosilicon was observed with the (3-mercaptopropyl)trimethoxysilane treatment, showing complete removal of lignin side chains, the lowest syringyl/guaiacyl ratio, depolymerization of cellulose and xylan, and reactivity with the C6 primary hydroxyls in cellulose. This may explain the outstanding stabilizing effectiveness of this silane and supports the conclusion that extensive chemical interactions are essential in this process. It also indicates the vital role of a mercapto group in wood stabilization by organosilicons. This 2D NMR technique sheds new light on the chemical mechanisms involved in organosilicon consolidation of wood and reveals what chemical characteristics are essential in developing future conservation treatments.

## 1. Introduction

The oldest known method for the conservation of waterlogged wooden artifacts dates back to the mid-1800s, when hot solutions of alum salts (KAl(SO_4_)_2_·12H_2_O) were used for this purpose for the first time [1,2]. More recent standard conservation procedures employ mainly polyethylene glycols of different molecular weights and various sugars [3,4,5,6,7,8,9]. However, since none of these methods has been entirely satisfying and some of them, such as alum and PEG treatment, turned out to be even detrimental to wooden artifacts in the long term [1,10,11,12,13,14], the search for more reliable solutions continues. One of the newly tested methods for waterlogged wood conservation is the application of organosilicon compounds, among which some (e.g., alkoxysilanes) can polymerize inside the wood structure, forming a stabilizing 3D network [15,16,17].

Our foregoing research on organosilicons allowed us to identify several compounds effective in stabilizing waterlogged wood dimensions during drying, including methyltrimethoxysilane, (3-mercaptopropyl)trimethoxysilane, (3-aminopropyl)triethoxysilane, 1,3-bis(3-aminopropyl)-1,1,3,3-tetramethyldisiloxane, or 1,3-bis-[(diethylamino)-3-(propoxy)propan-2-ol]-1,1,3,3-tetramethyldisiloxane [15,16]. They differ significantly in molecular weight, size, and chemical structure, which suggests different stabilizing mechanisms. Surprisingly, among the tested organosilicons that turned out ineffective in wood stabilization were some with a similar structure to those effective ones, differing only in the presence/absence of a particular side group or the length of the side chain. That indicates that not only the formation of a spatial network inside the wood tissue by polymerized organosilicon compounds but also that their chemical reactivity with wood polymers may contribute to their stabilizing efficiency.

There are several potential reactive sites in both wood polymers and organosilicon compounds that enable the formation of covalent or hydrogen bonds between their molecules. In wood polymers, they include primary (at **C6**) and secondary (at **C2** and **C3**) hydroxyls in cellulose [18,19], free hydroxyls present on all sugar units in hemicelluloses [20,21], and phenolic α-O-4 and β-O-4 linkages, as well as aliphatic and phenolic hydroxyl groups in lignin [22,23]. In turn, alkoxysilanes have highly reactive alkoxy groups that enable their polymerization by reacting with other silane molecules or different chemicals [24]. Additionally, organosilicons can contain several different functional groups, such as mercapto, thiocyanate, amine, vinyl, epoxy, etc., that may facilitate interactions with wood polymers [25,26].

Although it has already been confirmed that various silanes can react with cellulose [27,28,29,30,31,32,33], lignin [33,34,35,36,37,38], and wood [39,40,41,42,43], and the results of our previous FT-IR analyses on waterlogged wood treated with organosilicons confirmed the formation of new chemical bonds between them [15,43], the details of the interactions and potential preferences of silanes to react with individual wood polymers remain not fully understood. Therefore, to unveil the mechanism of waterlogged wood stabilization by organosilicon compounds, further research is necessary that will help to better understand the wood–silane interactions, especially in highly decayed wood where the usual cellulose/lignin ratio and the regular chemical composition and structure of wood polymers are altered by degradation processes.

One of the methods that provide insights into changes in wood chemistry caused by its modification is two-dimensional solution-state nuclear magnetic resonance (NMR) spectroscopy. The technique has been successfully employed to study the reactivity of wood polymers with various chemicals and modification agents, including phenol-formaldehyde adhesive [44], functionalized benzoic acids [45], polymeric methylene diphenyl diisocyanate [46], or N-methylimidazole (NMI) and acetic anhydride [47]. It is also helpful in qualitative and quantitative analyses of cell wall polymers in plant tissues [48], allows us to study of interactions between them [49], and facilitates the identification of structural changes in lignin, cellulose, and hemicelluloses caused by wood-decaying fungi [50,51] or hydrothermal pretreatment and enzymatic hydrolysis [52].

In the present study, the two-dimensional solution-state nuclear magnetic resonance (NMR) method was used to address four crucial questions: (1) whether any new chemical bonds are formed between organosilicons applied as wood consolidants and the cell wall polymers that remained in the degraded waterlogged wood; (2) which active sites in wood polymers interact with particular groups present in organosilicon molecules; (3) whether silanes have any preference to individual wood polymers; and, finally, (4) what makes an organosilicon an effective stabilizer (from the perspective of the chemical structure and reactivity). Understanding the interactions of organosilicons with wood polymers and the resulting wood stabilization mechanism will enable the design of more effective consolidants for waterlogged wood. It will also help develop new functional lignocellulosic materials modified with organosilicon compounds for different industrial purposes.

## 2. Results

### 2.1. Effectiveness of Organosilicon Compounds in Waterlogged Wood Stabilization

Keeping the original dimensions of waterlogged wooden artifacts during drying is a primary goal of successful conservation. Therefore, the effectiveness of conservation agents applied as waterlogged wood consolidants is usually evaluated based on parameters that measure dimensional wood stability, including shrinkage and anti-shrink efficiency [15,53,54].

Table 1 presents the efficacy of selected organosilicons used to stabilize highly degraded waterlogged archeological elm. Alkoxysilanes and siloxanes are labeled with the A and S letters, respectively, followed by the consecutive numbers (the full names of the chemicals are given in Section 4.1 Materials). The most effective wood-stabilizing treatment (with anti-shrink efficiency (ASE) over 80%) was that with (3-mercaptopropyl)trimethoxysilane (**A3**), 3-bis(3-aminopropyl)-1,1,3,3-tetramethyldisiloxane (**S2**), 1,3-bis-[(diethylamino)-3-(propoxy)propan-2-ol]-1,1,3,3-tetramethyldisiloxane (**S3**), 3-[3-(hydroxy)(polyethoxypropyl)]1,1,1,3,5,5,5-heptamethyltrisiloxane (**S7**), and methyltrimethoxysilane (**A1**). Pretty good stabilization with ASE over 70% was achieved using 1,3,5,7-tetrakis(1-(diethylamino)-3-(propoxy)propane-2-ol)-1,3,5,7-tetramethylcyclotetrasiloxane (**S5**) and (3-thiocyanatopropyl)trimethoxysilane NCS(CH_2_)_3_Si(OCH_3_)_3_ (**A4**). The other organosilicons used in the study were less effective, with ASE values of about 50%, which is insufficient from the conservation perspective.

Considering the general molecular structure (alkoxysilanes and siloxanes), the molecular weight, and the weight percent gain of the organosilicons applied (Table 1), it was difficult to determine any simple correlation between these parameters and the wood dimensional stabilization achieved with the treatment. Amongst the alkoxysilanes and siloxanes, the most effective were agents contained molecules as small as 136 g mol^−1^ and 196 g mol^−1^ (**A1** and **A3**), medium size of 248 g mol^−1^ (**S2**), or as large as 508 g mol^−1^ (**S3**) and 588 g mol^−1^ (**S7**). Weight percent gain in the range of 212% to 236% was obtained for both the most effective (**S2**, **S3**, **S7**, **A1**, **S5**, and **A4**) and less effective (**S1** and **S4**) chemicals. Interestingly, the best stabilizer (**A3**) was characterized by one of the smallest molecular weights (196 g mol^−1^) and the lowest *WPG* (only 137% g mol^−1^), too.

**Figure 1:**
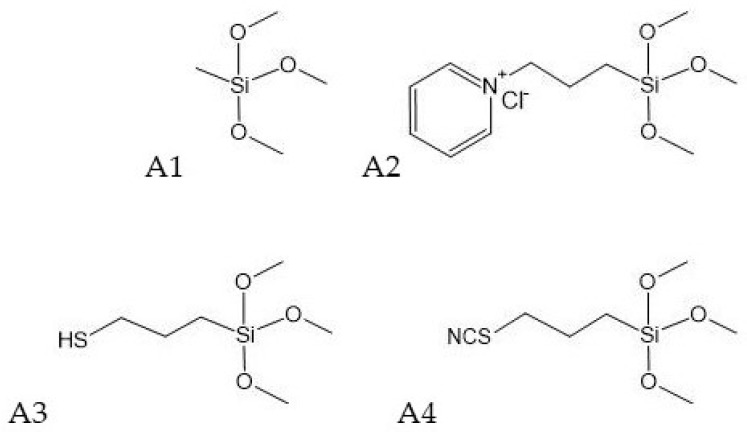
Chemical structures of alkoxysilanes used for waterlogged wood conservation.

Additionally, there was no direct correlation between the chemical structure and the stabilizing effectiveness of the organosilicon compounds used in this study. All alkoxysilanes (Figure 1) had a similar structure with the presence of three methoxyl groups. They differed only in the fourth group, which varied from a simple methyl group in A1 through a longer alkyl chain (propyl) terminated with a pyridinium chloride (**A2**), a thiol group (**A3**), or a thiocyanate group (**A4**). However, their waterlogged wood-stabilizing efficiency differed significantly (Table 1), which suggested the critical role of the fourth additional chemical group bound to the silicon atom.

**Figure 2:**
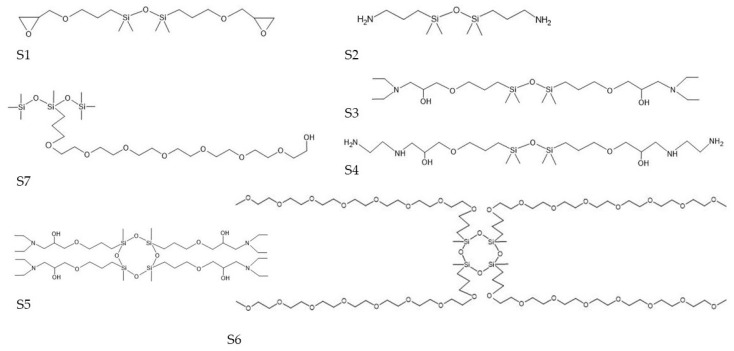
Chemical structures of siloxanes used for waterlogged wood conservation.

The structures of siloxanes used in the research were more varied (Figure 2). They included disiloxanes with shorter (**S1** and **S2**) and longer alkyl chains (**S3**, **S4**), with additional amino (**S2**, **S3**, **S4**) or epoxy (**S1**) groups, a trisiloxane with a long polyethoxypropyl chain (**S7**), as well as more complex cyclic tetrasiloxanes (**S5** and **S6**). Similarly to alkoxysilanes, it was difficult to find a correlation between the structure and stabilizing effectiveness of these chemicals because the best-performing ones (**S2**, **S3**, **S7**, and **S5**) differed in the length of a side chain and the presence of reactive groups, while some of the less effective ones had side chains of similar length and also contained a reactive group that could interact with wood polymers (e.g., **S1** vs. **S3** or **S4** vs. **S3**).

The results presented above suggest that more than one mechanism must be involved in the stabilizing effect of the organosilicon compounds on waterlogged wood. Hence the idea to use the two-dimensional (2D) ^1^H–^13^C single-bond correlation NMR technique to investigate the reactivity between organosilicons and wood polymers.

### 2.2. NMR Spectra

**Figure 3:**
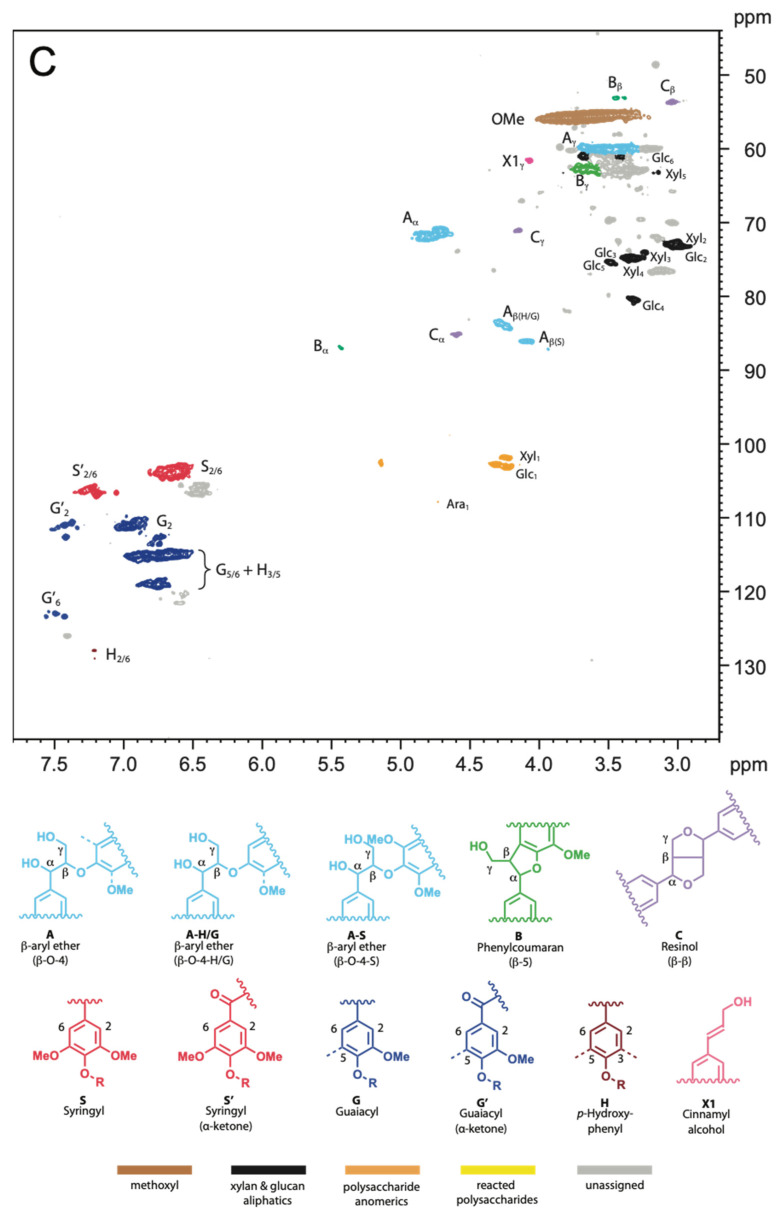
The partial 2D NMR spectrum of the control degraded archaeological elm (**C**) (**top**) and the chemical structures (**bottom**) of the main wood polymers present in the spectrum and the following figures.

**Figure 4:**
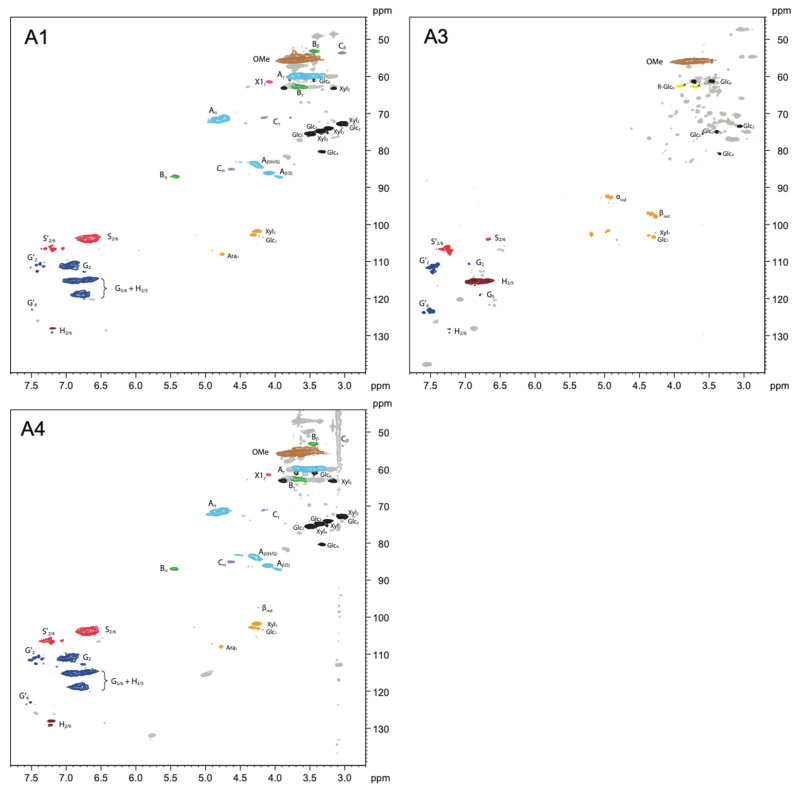
Partial 2D NMR spectra of alkoxysilane-treated wood showing the effects of the treatment with methyltrimethoxysilane (**A1**), (3-mercaptopropyl)trimethoxysilane (**A3**), and (3-thiocyanatopropyl)trimethoxysilane (**A4**). The colored contours and labels correspond to the chemical structures shown in Figure 3.

**Figure 5:**
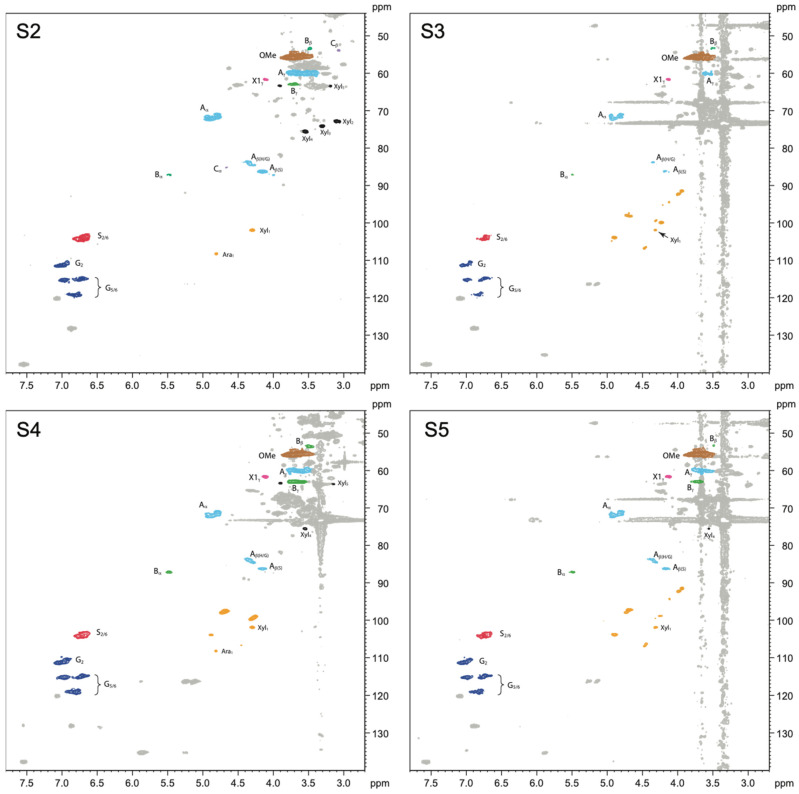
Partial 2D NMR spectra of siloxane-treated wood showing the effects of the treatment with (1,3-bis(3-aminopropyl)-1,1,3,3-tetramethyldisiloxane (**S2**), 1,3-bis-[(diethylamino)-3-(propoxy)propan-2-ol]-1,1,3,3-tetramethyldisiloxane (**S3**), 1,3-bis-[(ethylenodiamino)-3-(propoxy)propan-2-ol]-1,1,3,3-tetramethyldisiloxane (**S4**), 1,3,5,7-tetrakis(1-(diethylamino)-3-(propoxy)propan-2-ol)-1,3,5,7-tetramethylcyclotetrasiloxane (**S5**). The colored contours and labels correspond to the chemical structures shown in Figure 3.

**Figure 6:**
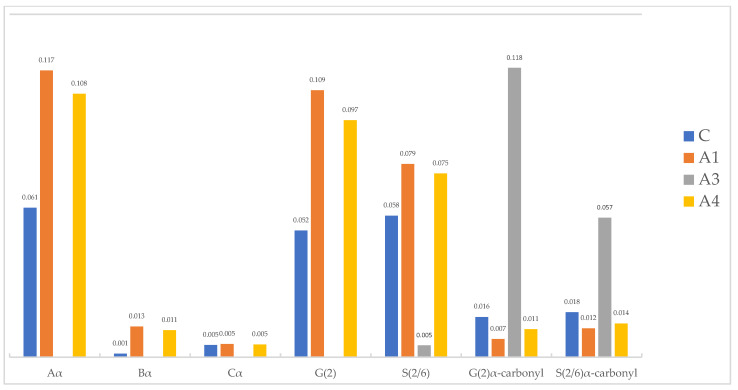
Bar chart summarizing the 2D NMR integrations of peaks from lignin subunits present in each spectrum of alkoxysilane-treated wood (**A1**, **A3**, and **A4**) with control degraded wood (**C**). The numbers above the bars indicate the actual value of the integral. All integrations are relative to the lignin methoxyl peak.

**Figure 7:**
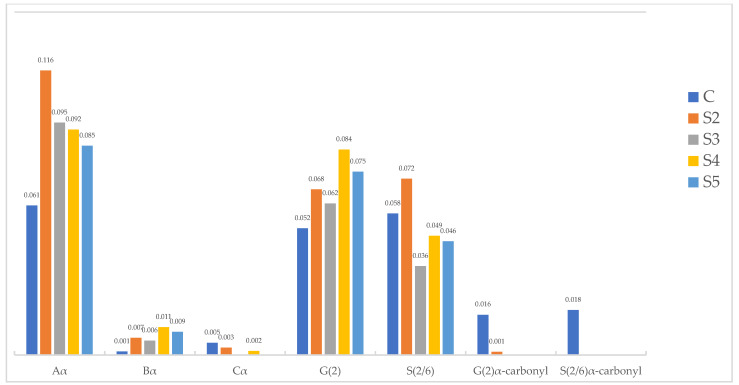
Bar chart summarizing the 2D NMR integrations of peaks from lignin subunits present in each spectrum of siloxane-treated wood (**S2**, **S3**, **S4**, and **S5**) with control degraded wood (**C**). The numbers above the bars indicate the actual value of the integral. All integrations are relative to the lignin methoxyl peak.

Two-dimensional (2D) ^1^H–^13^C single-bond correlation NMR spectra were acquired on the whole cell walls of alkoxysilane- and siloxane-treated archaeological elm wood. Through this analysis, the native wood cell wall polymers were semi-quantifiable, and the detailed chemistry between a treatment and the wood could be visualized, thus revealing clues as to the mechanisms involved in how each treatment stabilizes the wood. Figure 3, Figure 4 and Figure 5 display partial 2D NMR spectra for all samples studied here. Figure 3 includes a chemical structure key to the color-coded contours that are referenced in each spectrum. Figure 6 and Figure 7 are bar charts showing the NMR integration values for the major wood polymers present in each of the spectra relative to the lignin methoxyl group (for alkoxysilane- and siloxane-treated wood, respectively). Not all of the spectra displayed the presence of the major wood polymers due to the overwhelming intensities of the organosilicon contours. For example, the 2D NMR spectra of archaeological elm wood treated with **A2**, **S1**, **S6**, and **S7** showed intense organosilicon peaks that overlapped with the wood cell wall polymer peaks, making the signals from wood visually obscured. The organosilicon treatments may be grouped into two types: alkoxysilanes and siloxanes. The following will describe the chemical characteristics found in the wood cell walls after each treatment.

#### 2.2.1. Alkoxysilane-Treated Wood

Archaeological wood treated with alkoxysilanes included methyltrimethoxysilane (**A1**), 1-[3-(trimethoxysilyl)propyl]pyridinium chloride (**A2**), (3-mercaptopropyl)trimethoxysilane (**A3**), and (3-thiocyanatopropyl)trimethoxysilane (**A4**). The chemical structures for these treatments are shown in Figure 3.

Treatment A1 was the simplest structure of all the organosilicons. From Figure 4, the NMR spectrum showed quite similar characteristics to the control (**C**) degraded wood (Figure 3). For example, all the main lignin linkages (β-O-4, β-5, and β-β), the syringyl (S) and guaiacyl (G) units, and major polysaccharides cellulose (Glc) and xylan (Xyl) were present. The oxidized aromatic units were evidenced by the presence of α-carbonyl versions of syringyl and guaiacyl units, as depicted by S′ and G′. From the integration data shown in Figure 6, the S/G ratio of A1 decreased by 31% compared to the control.

Treatment A2 was the only organosilicon based on a salt. In the spectrum shown in Supplementary Figure S1, the major wood polymers were not able to be detected. The only functional group detectable from wood was that of the lignin methoxyl, and even this group was considered a weak signal. The high intensity of the treatment contours seemed to overwhelm the weaker wood polymer signals.

Treatment A3 contained a mercaptopropyl group. The NMR spectrum shown in Figure 4 displayed a dramatic degradation of the wood polymers. For example, the spectrum was devoid of all the major lignin linkages, as well as the predominant lignin aromatic units; the only aromatic units present were the α-carbonyl versions of syringyl and guaiacyl units (S′ and G′). The contour peak for *p*-hydroxyphenyl units (H) was shown to be enhanced, while the S and G contour peaks were depleted, showing evidence of methoxyl removal. Similarly, the major polysaccharides were also heavily cleaved; the α- and β-reducing end groups (α_red_ and β_red_) of cellulose (Glc) and xylan (Xyl) showed intense signals. Therefore, this treatment resulted in a high amount of wood degradation. However, we also detected partial reactivity between the treatment and the Glc_6_ position, showing new contours labeled R-Glc_6_ (yellow). These new contours showed evidence that this treatment does react with cellulose. In the integration data, shown in Figure 6, the S/G ratio of A3 decreased by 54% as compared to the control.

Treatment A4 contained a thiocyanatopropyl group. The NMR spectrum in Figure 4 displayed similar characteristics to spectra of the control degraded wood (Figure 3) and treatment A1. All of the major lignin linkages were present, as well as the aromatic lignin units (S and G). The α-carbonyl versions syringyl and guaiacyl units (S′ and G′) were also present. Cellulose and xylan were also evident. From Figure 6, the S/G ratio of A4 decreased by 27% compared to the control.

#### 2.2.2. Siloxane-Treated Wood

Archaeological wood treated with siloxanes included 1,3-bis(3-glycidyloxypropyl)-1,1,3,3-tetramethyldisiloxane (**S1**), 1,3-Bis(3-aminopropyl)-1,1,3,3-tetramethyldisiloxane (**S2**), 1,3-bis-[(diethylamino)-3-(propoxy)propan-2-ol]-1,1,3,3-tetramethyldisiloxane (**S3**), 1,3-bis-[(ethylenodiamino)-3-(propoxy)propan-2-ol]-1,1,3,3-tetramethyldisiloxane (**S4**), 1,3,5,7-tetrakis(1-(diethylamino)-3-(propoxy)propan-2-ol)-1,3,5,7-tetramethylcyclotetrasiloxane (**S5**), 1,3,5,7-tetrakis(3-polyethoxypropyl)-1,3,5,7-tetramethyltetracyclosiloxane, methoxy terminated (**S6**), and 3-[3-(hydroxy)(polyethoxypropyl)]1,1,1,3,5,5,5-heptamethyltrisiloxane (**S7**). The chemical structures for these treatments are shown in Figure 2.

In all NMR spectra obtained for wood treated with siloxanes of the molecular weight over 300 g mol^−1^ (**S1**, **S3**–**7**), the high-intensity peaks coming from the treatments that overwhelmed the wood polymers signals were seen.

Treatments **S1**, **S2**, **S3**, and **S4** all contained a tetramethyldisiloxane group. Treatments **S2**, **S3**, and **S4** showed similar characteristics in their NMR spectra (Figure 5). For example, the major lignin linkages β-O-4 and β-5 were present as well as the S and G units. However, the S′ and G′ units were absent, which suggests the reduction or removal of the α-carbonyl functionality. From the integration data, shown in Figure 7, the S/G ratios for treatments **S2**, **S3**, and **S4** all showed a decrease of 7%, 47%, and 48%, respectively, compared to the control. The polysaccharides also showed several different contour peaks as compared to the control, suggesting reactivity between the treatment and the polysaccharides, especially for treatments **S3** and **S4**. Treatment **S1** was devoid of major wood polymer peaks, most likely due to the overwhelming peaks from the organosilicon treatment, but did show the presence of polysaccharide peaks in the anomeric region and S units in the aromatic region (Figure S1).

Treatments **S5** and **S6** both contained a tetramethyltetracyclosiloxane group. Interestingly, treatment **S5** displayed similar characteristics to treatments **S3** and **S4** in the NMR spectra in that the major lignin linkages β-O-4 and β-5 were present, as well as the S and G units, and the polysaccharide peaks looked similar in the anomeric region (Figure 5). On the other hand, treatment **S6** was devoid of major lignin polymer peaks, most likely due to the overwhelming peaks from the organosilicon treatment, but did show polysaccharide peaks in the anomeric region (Figure S1). From Figure 7, the S/G ratio for treatment **S5** showed a decrease of 45% compared to the control

Treatment **S7** contained a heptamethyltrisiloxane group. Similar to treatment **S6** characteristics, this treatment was also mostly devoid of wood polymer peaks with the exception of weak aromatic units (S and G) and polysaccharide peaks in the anomeric region (Figure S1). The S/G ratio was not measurable, given the weak intensity of the aromatic unit peaks.

## 3. Discussion

The NMR results obtained shed new light on the interactions between the wood cell wall polymers and organosilicon compounds applied as consolidants to stabilize waterlogged wood dimensions.

Our previous FT-IR studies [15,43] showed that hydrolysis and condensation of alkoxysilane monomers occurred in the treated wood, leading to the formation of a stabilizing polymer network inside the wood structure. Moreover, it seemed that also new chemical bonds between wood hydroxyls and alkoxysilanes were formed due to the treatment, in particular when methyltrimethoxysilane (**A1**) and (3-mercaptopropyl)trimethoxysilane (**A3**) were applied. The reduction of available hydroxyls on the cell walls was additionally confirmed by dynamic water sorption experiments that showed the decrease in equilibrium moisture content and the sorption hysteresis of treated archaeological wood compared to untreated wood [55,56]. However, the new NMR data only showed the chemical modification of C6 primary hydroxyls in cellulose in the wood sample treated with **A3**; no other evidence of wood hydroxyls’ modification is visible.

On the other hand, we observed demethoxylation of lignin S and G units, which was also seen in our previous FT-IR spectra [43] and other studies on wood modification with alkoxysilanes [57,58]. From the integration data in Figure 6, it was evident that the major lignin linkages in all samples treated with alkoxysilanes were much—in some cases two times—higher than that found in the control degraded wood samples, with the exception of the β-β linkage. This result, in conjunction with the overwhelmingly consistent decrease in the S/G ratios in the treated woods compared to the control, suggests that the lignin was undergoing demethoxylation during alkoxysilane treatment; thus, alkoxysilanes have the ability to oxidize these methoxyl groups on the aromatic ring of lignin.

The NMR results were, then, surprising, especially since several researchers observed the reactivity of alkoxysilanes (via alkoxy groups) with wood hydroxyls [24,39,41,58]. However, some catalysts are usually applied to promote the reactivity of alkoxysilanes with wood, which were not used in the case of our waterlogged wood treatments. This fact may explain why we did not observe the modification of the wood hydroxyls in our NMR spectra of **A1**, **A2**, and **A4**. It is worth mentioning here that the NMR spectra obtained contained some unidentified peaks that arose from the alkoxysilanes applied. For example, in the spectra shown in Figure 4 we do see peaks from the trimethoxy groups around 3.0–3.5/45–50 ppm; most of the other peaks from alkoxysilanes did not interfere with wood polymer peaks. However, we cannot currently assign any other peaks related to new plausible chemical bonds between the silanes and lignin/polysaccharides units in **A1**, **A2**, and **A4**. Further research on this phenomenon is planned.

For alkoxysilane treatment **A3** ((3-mercaptopropyl)trimethoxysilane), we were able to tentatively assign the new peaks of reacted cellulose **C6** hydroxyls, labeled as R-Glc_6_, at 3.73/63.1 ppm and 3.93/63.1 ppm (Figure 4). Treatment **A3** also showed complete removal of lignin side chains, the lowest S/G ratio (0.52), and depolymerization of cellulose and xylan as evidenced by the presence of their intensified α- and β-reducing end groups at 4.97/92.8, 4.93/93.0 and 4.34/97.5, 4.28/98.3 ppm, respectively. This strong reactivity of the most effective wood stabilizer with all wood polymers indicates that chemical interactions are essential in stabilizing waterlogged wood dimensions during drying. It also points to the conclusion about a vital role the mercapto group plays in these interactions and wood stabilization. Even though the observed reactivity may hinder the reversibility of the treatment with this silane, which is required by conservation ethics, the SEM images of the treated wood showed that the silane locates in/on the cell wall [15], leaving the lumina empty for further re-treatment, which potentially does not exclude the chemical from the conservation practice.

Interestingly, in the case of siloxane treatment, we could mainly observe lignin modification employing demethoxylation of S and G units and removal (decarboxylation) of the α-carbonyl versions of syringyl and guaiacyl units (S′ and G′). From the integration data in Figure 7, as with what we observed for the alkoxysilane treatments, the major lignin linkages in all of the samples treated with siloxanes were much higher than those found in control degraded wood samples, with the exception of the β-β linkage. This result suggests that the lignin can undergo demethoxylation during organosilicon treatment, regardless of treatment type. From our previous FT-IR research [43], we learned that the methoxyl groups in lignin might contribute to the interaction with 1,3-bis-[(diethylamino)-3-(propoxy)propan-2-ol]-1,1,3,3-tetramethyldisiloxane (**S3**). Perhaps all siloxanes applied in this study react with lignin similarly. However, considering their diverse effectiveness as wood consolidants, this reactivity seems not to play a crucial role in the stabilizing mechanism.

When it comes to decarbonylation, the literature data indicate that siloxanes, including tetramethyldisiloxane, can reduce α,β-unsaturated carbonyl derivatives [59,60]. A tetramethyldisiloxane unit is present in all siloxanes used in our research. However, it contains methyl groups attached to the silicon atom instead of hydrogen, which is necessary for the reductive activity. Therefore, it is difficult to say if the treatment conditions or the solvent used for NMR analysis (DMSO) could cause demethylation of the silicon atom and foster the reductive properties of siloxanes; this question requires further study to be answered.

Another aspect that was evident from the NMR analysis of siloxane-treated wood was the absence of the native cellulose anomeric peak normally observed at 4.25/103.7 ppm. Cellulose was clearly present in all of the alkoxysilane-treated wood samples; so, this indicates that the siloxane treatments are able to modify cellulose heavily. Several new anomeric peaks were observed in the siloxane spectra of S3, S4, and S5 (Figure 5) and **S1**, **S6**, and **S7** (Figure S1), but were not currently assigned here. Further research is needed to assign these new anomeric peaks. Since the effectiveness of the applied siloxanes varied, it is difficult to explicitly state if polysaccharide modification is essential in stabilizing waterlogged wood dimensions during drying.

Considering the results of the presented research and the data on organosilicon compounds used for waterlogged wood conservation from the previous studies [15,16,43], it can be concluded that the mechanism of waterlogged archaeological wood dimensional stabilization by alkoxysilanes is based on (1) bulking the cell wall, (2) forming a stabilizing polymer network, and (3) chemically interacting with wood polymers (at least in the case of alkoxysilanes containing mercapto groups). On the other hand, in the case of siloxanes, wood stabilization seems to be mainly based on filling the cell lumina. However, the absence of the native cellulose anomeric peak and the absence of the α-carbonyls in aromatic lignin units in all the siloxane treatments demonstrate that cellulose and lignin modifications are also intimately involved in stabilizing siloxane-treated wood.

## 4. Materials and Methods

### 4.1. Materials

The research material was waterlogged elm (*Ulmus* spp.) heartwood: the remnants of a medieval bridge excavated from the sediments of the Lednica Lake in the Wielkopolska Region, Poland. The wood was highly degraded, with reduced cellulose and hemicelluloses content and the loss of wood substance estimated at about 70–80% [15,43].

Organosilicon compounds for waterlogged wood treatment were obtained by hydrosilylation of relevant olefins with Si–H-containing compounds in the presence of platinum catalysts [61] at the Adam Mickiewicz University Foundation, Poznań Science and Technology Park, Poznań, Poland [15]:

- methyltrimethoxysilane CH_3_Si(OCH_3_)_3_ (**A1**);
- 1-[3-(trimethoxysilyl)propyl]pyridinium chloride (C_5_H_5_NCl)C_3_H_6_Si(OCH_3_)_3_ (**A2**);
- (3-mercaptopropyl)trimethoxysilane HS(CH_2_)_3_Si(OCH_3_)_3_ (**A3**);
- (3-thiocyanatopropyl)trimethoxysilane NCS(CH_2_)_3_Si(OCH_3_)_3_ (**A4**);
- 1,3-bis(3-glycidyloxypropyl)-1,1,3,3-tetramethyldisiloxane [CH_2_(O)CHCH_2_O(CH_2_)_3_Si(CH_3_)_2_]_2_O (**S1**);
- 1,3-bis(3-aminopropyl)-1,1,3,3-tetramethyldisiloxane [H_2_N(CH_2_)_3_Si(CH_3_)_2_]_2_O (**S2**);
- 1,3-bis-[(diethylamino)-3-(propoxy)propan-2-ol]-1,1,3,3-tetramethyldisiloxane [(C_2_H_5_)_2_NCH_2_CH(OH)CH_2_O(CH_2_)_3_Si(CH_3_)_2_]_2_O (**S3**);
- 1,3-bis-[(ethylenodiamino)-3-(propoxy)propan-2-ol]-1,1,3,3-tetramethyldisiloxane [(NH_2_(CH2)_2_HNCH_2_CH(OH)CH_2_O(CH_2_)_3_Si(CH_3_)_2_]_2_O (**S4**);
- 1,3,5,7-tetrakis(1-(diethylamino)-3-(propoxy)propan-2-ol)-1,3,5,7-tetramethylcyclotetrasiloxane [(C_2_H_5_)_2_NCH_2_CH(OH)CH_2_O(CH_2_)_3_Si(CH_3_)O]_4_ (**S5**);
- 1,3,5,7-tetrakis(3-polyethoxypropyl)-1,3,5,7-tetramethyltetracyclosiloxane methoxy terminated [CH_3_O(CH_2_CH_2_O)_7_(CH_2_)_3_Si(CH_3_)O]_4_ (**S6**);
- 3-[3-(hydroxy)(polyethoxypropyl)]1,1,1,3,5,5,5-heptamethyltrisiloxane HO(CH_2_CH_2_O)_7_(CH_2_)_3_Si(CH_3_)[OSi(CH_3_)_3_]_2_ (**S7**).

For simplicity, the numbers of consecutive alkoxysilanes (**A**) and siloxanes (**S**) listed above, instead of their full chemical names, are used throughout the manuscript.

Dimethylsulfoxide-d_6_ (DMSO-d_6_, 99.5% D) and 1-methylimidazole-d_6_ (NMI-d_6_) for NMR analysis were supplied by Aldrich Chemical Company (Milwaukee, WI, USA).

### 4.2. Methods

#### 4.2.1. Waterlogged Wood Treatment

Waterlogged elm log was cut into small samples with the dimensions of 20 mm × 20 mm × 10 mm (radial × tangential × longitudinal direction). To ensure the greatest possible homogeneity of the wood degradation degree, thus reproducibility of the results, the specimens were sampled from a selected part of the log and a similar distance from the pit, since the number of suitable wooden pieces was limited.

The specimens were dehydrated by soaking them in 96% ethanol for 4 weeks and then treated with 50% ethanol solutions of selected organosilicon compounds. An oscillating-pressure method was used for the wood treatment, applying a −0.9 bar vacuum for 0.5 h and then 10 bars of pressure for 6 h. The cycle was repeated six times every 24 h. Between the cycles, the wood was left submerged in the organosilicon solution under atmospheric pressure to ensure continuous treatment. After the treatment, the samples were removed from the conservation solution and air-dried at room temperature (21 ± 1 °C) for 4 weeks. As a result, five replicates of each treatment were obtained, and five more untreated specimens were air-dried from the waterlogged state and used as a standard control for this type of wood.

To evaluate the effectiveness of the treatment, weight percent gain (*WPG*) for each organosilicon compound was calculated according to the standard Equation (1):

(1) $$WPG=\frac{W_{1}-W_{0}}{W_{0}}\times 100$$

where $W_{0}$ is the estimated dry mass of the specimen before treatment, and $W_{1}$ is the dry mass of the sample treated with a selected organosilicon compound [15].

The evaluation of the stabilizing effect of particular conservation agents was based on the values of volumetric shrinkage ($S_{v}$) and volumetric anti-shrink efficiency coefficient ($ASE_{v}$) calculated according to the standard Equations (2) and (3):

(2) $$S_{v}=\frac{V_{0}-V_{1}}{V_{0}}\times 100$$

where $V_{0}$ is the initial volume of a waterlogged specimen, and $V_{1}$ is the final volume of the specimen (untreated or treated, respectively) after air-drying, and

(3) $$ASE_{v}=\frac{S_{vu}-S_{vt}}{S_{vu}}\times 100$$

where $S_{vu}$ is the volumetric shrinkage of the untreated specimen, and $S_{vt}$ is the volumetric shrinkage of the treated specimen.

#### 4.2.2. Wood Preparation for NMR

Air-dried archaeological wood samples were sliced with a knife in the radial direction to obtain sections approximately 1 mm thick. Each sliced specimen was placed into a 50 mL ZrO_2_ jar followed by three 20 mm ZrO_2_ balls and loaded into a Retsch PM-400 planetary ball mill (Newtown, PA, USA). The wood was milled for 24 h (300 rpm, 20 min milling, 10 min pause; these conditions allowed us to keep the wood temperature below 50 °C, which prevented its thermal degradation and changes in the chemical composition). Then, the 20 mm balls were removed and shaken in the copper sieve to recover the wooden material that remained on their surface. Afterward, ten 10 mm ZrO_2_ were added to pre-milled wood, and the milling was continued for another 24 h under the same conditions. Following ball-milling, the 10 mm balls were removed and shaken in the copper sieve, as performed previously, and the milled wood was scraped from the jar and weighed.

#### 4.2.3. Wood Cell Wall Dissolution

About 30 mg of each sample was placed in the NMR tube (5 mm in diameter, 17.8 cm in length) and dissolved using 400 µL DMSO-d_6_. To expedite the wood dissolution, the tubes were sonicated at 35 °C for 1 h [62]. For the samples that dissolved successfully, giving homogeneous and transparent solutions, an additional 100 µL DMSO-d_6_ was added to reach the final solvent volume of 500 µL. For the samples that did not fully dissolve, 50 µL of 1-methylimidazole-d_6_ (NMI-d_6_), a non-degradative co-solvent with DMSO-d_6_, was added to facilitate the disruption of hydrogen bonds. NMI-d_6_ was added to samples **A2**, **A3**, **S1**, **S2**, **S3**, **S4**, **S5**, **S6**, and **S7**; it was omitted in samples **C**, **A1**, and **A4** because dissolution proceeded in DMSO-d_6_ without the need for NMI-d_6_. Adding NMI-d_6_ as a co-solvent did not affect the NMR chemical shifts of the wood cell wall polymers in this study. Then, the samples were sonicated until homogeneous and clear solutions evolved. In the end, 50 µL DMSO-d_6_ was added to the tubes with NMI-d_6_ to reach the final solvent volume of 500 µL.

#### 4.2.4. NMR Analysis

NMR spectra for untreated and treated archaeological wood were acquired using a Bruker-Biospin (Rheinstetten, Germany) AVANCE III HD^TM^ 500 MHz spectrometer fitted with a nitrogen-cooled 5 mm Prodigy^TM^ TCI gradient cryoprobe with inverse geometry. The one-bond ^1^H–^13^C correlation (HSQC) spectra were obtained using the adiabatic Bruker pulse program hsqcetgpsisp2.2 and processed as previously described [63]. For semi-quantitative analysis of the wood polymer structures present in the spectra, specific chemical shifts of native structural units, such as the β-aryl ether, phenylcoumaran, and resinol subunits in lignin or arabinoxylan and glucomannan units in hemicellulose were integrated and referenced to the lignin methoxyl group (since it is known as the most stable functional group) using Bruker TopSpin 3.6.2 software.

## 5. Conclusions

The conservation of ancient wooden artifacts is critical in preserving history and retelling stories that would otherwise be lost. Developing unique and effective methods in wood conservation requires an understanding of the mechanisms involved in stabilizing the wood. Organosilicons have been proven to be highly effective as wood stabilizers. Here, we explored and characterized the detailed chemistry occurring between organosilicon treatments and the wood cell wall polymers using 2D ^1^H–^13^C solution-state NMR. The results of this study on the reactivity of organosilicon compounds applied as consolidants for waterlogged archaeological wood with wood cell wall polymers revealed an extensive modification of lignin and polysaccharides due to the treatment. In the case of alkoxysilanes, mainly lignin demethoxylation was observed. However, in the case of (3-mercaptopropyl)trimethoxysilane treatment, which was the most effective in stabilizing wood dimensions, more comprehensive interactions with wood polymers were observed, including depolymerization of cellulose and xylan, reactivity with the C6 primary hydroxyls in cellulose, complete removal of lignin side chains, and the lowest syringyl/guaiacyl unit ratio. In turn, siloxane treatments caused severe modification of lignin aromatics, including its α-decarboxylation and demethoxylation, as well as cellulose modification.

In answering the questions presented in our research objectives, we can state that:

- New chemical bonds were formed between (3-mercaptopropyl)trimethoxysilane and cellulose in waterlogged wood. In the case of other organosilicons, it was difficult to assign unidentified peaks in NMR spectra to potential new bonds formed between them and wood polymers. This problem is planned to be solved in future research.
- The active sites in wood polymers that interacted with organosilicons were **C6** primary hydroxyls in cellulose (in the case of (3-mercaptopropyl)trimethoxysilane treatment), as well as methoxyl (in both types of organosilicon treatments) and α-carbonyl groups in aromatic lignin units (in the case of siloxane treatment).
- In general, alkoxysilanes appear to preferentially react with lignin, while siloxanes can modify lignin and polysaccharides; only (3-mercaptopropyl)trimethoxysilane was confirmed to react also with cellulose.
- Since a similar modification of wood polymers was observed for both groups of organosilicons used in this study, but their effectiveness as wood stabilizers was different, we cannot state if lignin demethoxylation or modification of lignin aromatics by organosilicons plays a crucial role in the stabilizing mechanism; on the other hand, we can clearly state that the extensive chemical modification of 3-mercaptopropyl)trimethoxysilane (containing a reactive mercapto group) with wood polymers is crucial for the excellent stabilization of waterlogged wood dimensions during drying.

## Figures and Tables

**Table 1:** The parameters measured/calculated for selected organosilicons or wood samples treated with them: *S_v_*, volumetric wood shrinkage; *ASE_v_*, anti-shrink efficiency of the individual organosilicon compound; *WPG*, weight percent gain; MW, molecular weight of an organosilicon monomer; C, untreated waterlogged wood used as a control; the full names of organosilicon compounds are given in Section 4.1 Materials.

Organosilicon Applied	*S_v_* [%]	*ASE_v_* [%]	MW [g/mol]	*WPG* [%]
**C**	55.1 ± 4.9	-	-	-
**A1**	9.7 ± 1.3	82.4	136.22	231.9 ± 6.8
**A2**	29.2 ± 7.3	47.0	277.82	328.8 ± 1.3
**A3**	0.7 ± 0.5	98.7	196.34	136.9 ± 9.4
**A4**	15.9 ± 3.5	71.1	221.35	212.5 ± 1.9
**S1**	24.9 ± 1.7	54.8	362.61	227.3 ± 0.5
**S2**	4.5 ± 1.4	91.8	248.51	236.2 ± 1.9
**S3**	4.5 ± 1.4	91.8	508.88	219.8 ± 5.9
**S4**	29.7 ± 2.5	46.1	482.80	234.1 ± 2.2
**S5**	15.0 ± 0.9	72.8	989.62	231.8 ± 1.3
**S6**	26.3 ± 1.1	52.3	1762.40	270.8 ± 2.1
**S7**	5.3 ± 2.5	90.4	588.95	227.3 ± 1.4

## Data Availability

The data presented in this study are available on request from the corresponding author.

## Supplementary Materials

The following supporting information can be downloaded at https://www.mdpi.com/article/10.3390/molecules27113407/s1. Figure S1: Partial 2D NMR spectra of organosilicon-treated wood showing 1-[3-(trimethoxysilyl)propyl]pyridinium chloride (**A2**), 1,3-bis(3-glycidyloxypropyl)-1,1,3,3-tetramethyldisiloxane (**S1**), 1,3,5,7-tetrakis(3-polyethoxypropyl)-1,3,5,7-tetramethyltetracyclosiloxane methoxy terminated (**S6**), 3-[3-(hydroxy)(polyethoxypropyl)]1,1,1,3,5,5,5-heptamethyltrisiloxane (**S7**). The colored contours and labels correspond to the chemical structures shown in Figure 3. Table S1: Chemical shifts of functional groups present in selected organosilicon compounds used in this study. References [64,65,66,67,68,69,70,71,72,73] are cited in Supplementary Materials.

## References

[1] Braovac S., Kutzke H. (2012). The Presence of Sulfuric Acid in Alum-Conserved Wood–Origin and Consequences. J. Cult. Herit..

[2] Braovac S., Tamburini D., Lucejko J.J., McQueen C., Kutzke H., Colombini M.P. (2016). Chemical Analyses of Extremely Degraded Wood Using Analytical Pyrolysis and Inductively Coupled Plasma Atomic Emission Spectroscopy. Microchem. J..

[3] Hoffmann P. (1986). On the Stabilization of Waterlogged Oakwood with PEG. II. Designing a Two-Step Treatment for Multi-Quality Timbers. Stud. Conserv..

[4] Hoffmann P. (1990). On the Stabilization of Waterlogged Softwoods with Polyethylene Glycol (PEG). Four Species from China and Korea. Holzforschung.

[5] Hocker E., Almkvist G., Sahlstedt M. (2012). The Vasa Experience with Polyethylene Glycol: A Conservator’s Perspective. J. Cult. Herit..

[6] Collett H., Bouville F., Giuliani F., Schofield E. (2021). Structural Monitoring of a Large Archaeological Wooden Structure in Real Time, Post PEG Treatment. Forests.

[7] Tahira A., Howard W., Pennington E.R., Kennedy A. (2017). Mechanical Strength Studies on Degraded Waterlogged Wood Treated with Sugars. Stud. Conserv..

[8] Morgós A., Imazu S., Ito K. Sugar Conservation of Waterlogged Archaeological Finds in the Last 30 Years. Proceedings of the 2015 Conservation and Digitalization Conference.

[9] Hoffmann P. (2001). To Be and to Continue Being a Cog: The Conservation of the Bremen Cog of 1380. Int. J. Naut. Archaeol..

[10] Broda M., Hill C.A.S. (2021). Conservation of Waterlogged Wood–Past, Present and Future Perspectives. Forests.

[11] McQueen C.M., Tamburini D., Lucejko J.J., Braovac S., Gambineri F., Modugno F., Colombini M.P., Kutzke H. (2017). New Insights into the Degradation Processes and Influence of the Conservation Treatment in Alum-Treated Wood from the Oseberg Collection. Microchem. J..

[12] McQueen C.M.A., Mortensen M.N., Caruso F., Mantellato S., Braovac S. (2020). Oxidative Degradation of Archaeological Wood and the Effect of Alum, Iron and Calcium Salts. Herit. Sci..

[13] Mortensen M.N., Egsgaard H., Hvilsted S., Shashoua Y., Glastrup J. (2007). Characterisation of the Polyethylene Glycol Impregnation of the Swedish Warship Vasa and One of the Danish Skuldelev Viking Ships. J. Archaeol. Sci..

[14] Mortensen M.N., Egsgaard H., Hvilsted S., Shashoua Y., Glastrup J. (2012). Tetraethylene Glycol Thermooxidation and the Influence of Certain Compounds Relevant to Conserved Archaeological Wood. J. Archaeol. Sci..

[15] Broda M., Dąbek I., Dutkiewicz A., Dutkiewicz M., Popescu C.-M., Mazela B., Maciejewski H. (2020). Organosilicons of Different Molecular Size and Chemical Structure as Consolidants for Waterlogged Archaeological Wood–a New Reversible and Retreatable Method. Sci. Rep..

[16] Broda M., Mazela B., Dutkiewicz A. (2019). Organosilicon Compounds with Various Active Groups as Consolidants for the Preservation of Waterlogged Archaeological Wood. J. Cult. Herit..

[17] Kavvouras P.K., Kostarelou C., Zisi A., Petrou M., Moraitou G. (2009). Use of Silanol-Terminated Polydimethylsiloxane in the Conservation of Waterlogged Archaeological Wood. Stud. Conserv..

[18] Hidayat B.J., Felby C., Johansen K.S., Thygesen L.G. (2012). Cellulose Is Not Just Cellulose: A Review of Dislocations as Reactive Sites in the Enzymatic Hydrolysis of Cellulose Microfibrils. Cellulose.

[19] Heinze T., Rojas O.J. (2016). Cellulose: Structure and Properties. Cellulose Chemistry and Properties: Fibers, Nanocelluloses and Advanced Materials.

[20] Peng F., Ren J.L., Xu F., Sun R.-C. (2011). Chemicals from Hemicelluloses: A Review. Sustainable Production of Fuels, Chemicals, and Fibers from Forest Biomass.

[21] Sun R., Sun X.F., Tomkinson J. (2004). Hemicelluloses and Their Derivatives. ACS Symp. Ser..

[22] Varila T., Romar H., Luukkonen T., Hilli T., Lassi U. (2020). Characterization of Lignin Enforced Tannin/Furanic Foams. Heliyon.

[23] Antonino L.D., Gouveia J.R., de Sousa Júnior R.R., Garcia G.E.S., Gobbo L.C., Tavares L.B., dos Santos D.J. (2021). Reactivity of Aliphatic and Phenolic Hydroxyl Groups in Kraft Lignin towards 4,4′ MDI. Molecules.

[24] Donath S., Militz H., Mai C. (2004). Wood Modification with Alkoxysilanes. Wood Sci. Technol..

[25] Tesoro G. (1991). Yulong Wu Silane Coupling Agents: The Role of the Organofunctional Group. J. Adhes. Sci. Technol..

[26] Tingaut P., Weigenand O., Mai C., Militz H., Sèbe G. (2006). Chemical Reaction of Alkoxysilane Molecules in Wood Modified with Silanol Groups. Holzforschung.

[27] Brochier Salon M.-C., Abdelmouleh M., Boufi S., Belgacem M.N., Gandini A. (2005). Silane Adsorption onto Cellulose Fibers: Hydrolysis and Condensation Reactions. J. Colloid Interface Sci..

[28] de Oliveira Taipina M., Ferrarezi M.M.F., Yoshida I.V.P., Gonçalves M.D.C. (2013). Surface Modification of Cotton Nanocrystals with a Silane Agent. Cellulose.

[29] Nakatani H., Iwakura K., Miyazaki K., Okazaki N., Terano M. (2011). Effect of Chemical Structure of Silane Coupling Agent on Interface Adhesion Properties of Syndiotactic Polypropylene/Cellulose Composite. J. Appl. Polym. Sci..

[30] Salon M.-C.B., Gerbaud G., Abdelmouleh M., Bruzzese C., Boufi S., Belgacem M.N. (2007). Studies of Interactions between Silane Coupling Agents and Cellulose Fibers with Liquid and Solid-State NMR. Magn. Reson. Chem..

[31] Robles E., Csóka L., Labidi J. (2018). Effect of Reaction Conditions on the Surface Modification of Cellulose Nanofibrils with Aminopropyl Triethoxysilane. Coatings.

[32] Neves R.M., Ornaghi H.L., Zattera A.J., Amico S.C. (2020). The Influence of Silane Surface Modification on Microcrystalline Cellulose Characteristics. Carbohydr. Polym..

[33] Siuda J., Perdoch W., Mazela B., Zborowska M. (2019). Catalyzed Reaction of Cellulose and Lignin with Methyltrimethoxysilane—FT-IR, 13C NMR and 29Si NMR Studies. Materials.

[34] Prasetyo E.N., Kudanga T., Fischer R., Eichinger R., Nyanhongo G.S., Guebitz G.M. (2012). Enzymatic Synthesis of Lignin–Siloxane Hybrid Functional Polymers. Biotechnol. J..

[35] Kabir M.M., Wang H., Lau K.T., Cardona F. (2013). Effects of Chemical Treatments on Hemp Fibre Structure. Appl. Surf. Sci..

[36] Li H., Bunrit A., Li N., Wang F. (2020). Heteroatom-Participated Lignin Cleavage to Functionalized Aromatics. Chem. Soc. Rev..

[37] Zhang J., Chen Y., Brook M.A. (2014). Reductive Degradation of Lignin and Model Compounds by Hydrosilanes. ACS Sustain. Chem. Eng..

[38] Zhu J., Xue L., Wei W., Mu C., Jiang M., Zhou Z. (2015). Modification of Lignin with Silane Coupling Agent to Improve the Interface of Poly(L-Lactic) Acid/Lignin Composites. BioResources.

[39] Baur S.I., Easteal A.J. (2013). Improved Photoprotection of Wood by Chemical Modification with Silanes: NMR and ESR Studies. Polym. Adv. Technol..

[40] Wang Q., Xiao Z., Wang W., Xie Y. (2015). Coupling Pattern and Efficacy of Organofunctional Silanes in Wood Flour-Filled Polypropylene or Polyethylene Composites. J. Compos. Mater..

[41] Sèbe G., Tingaut P., Safou-Tchiama R., Pétraud M., Grelier S., Jéso B.D. (2004). Chemical Reaction of Maritime Pine Sapwood (Pinus Pinaster Soland) with Alkoxysilane Molecules: A Study of Chemical Pathways. Holzforschung.

[42] Grubbström G., Holmgren A., Oksman K. (2010). Silane-Crosslinking of Recycled Low-Density Polyethylene/Wood Composites. Compos. Part A Appl. Sci..

[43] Popescu C.-M., Broda M. (2021). Interactions between Different Organosilicons and Archaeological Waterlogged Wood Evaluated by Infrared Spectroscopy. Forests.

[44] Yelle D.J., Ralph J. (2016). Characterizing Phenol–Formaldehyde Adhesive Cure Chemistry within the Wood Cell Wall. Int. J. Adhes. Adhes..

[45] Namyslo J.C., Drafz M.H.H., Kaufmann D.E. (2021). Durable Modification of Wood by Benzoylation—Proof of Covalent Bonding by Solution State NMR and DOSY NMR Quick-Test. Polymers.

[46] Yelle D.J., Ralph J., Frihart C.R. (2011). Delineating PMDI Model Reactions with Loblolly Pine via Solution-State NMR Spectroscopy. Part 2. Non-Catalyzed Reactions with the Wood Cell Wall. Holzforschung.

[47] Lu F., Ralph J. (2003). Non-Degradative Dissolution and Acetylation of Ball-Milled Plant Cell Walls: High-Resolution Solution-State NMR. Plant J..

[48] Yelle D.J. (2020). Multifaceted Approach for Determining the Absolute Values for Lignin Subunits in Lignocellulosic Materials.

[49] Miyagawa Y., Tobimatsu Y., Lam P.Y., Mizukami T., Sakurai S., Kamitakahara H., Takano T. (2020). Possible Mechanisms for the Generation of Phenyl Glycoside-Type Lignin–Carbohydrate Linkages in Lignification with Monolignol Glucosides. Plant J..

[50] Yelle D.J., Ralph J., Lu F., Hammel K.E. (2008). Evidence for Cleavage of Lignin by a Brown Rot Basidiomycete. Environ. Microbiol..

[51] Yelle D.J., Kapich A.N., Houtman C.J., Lu F., Timokhin V.I., Fort Jr R.C., Ralph J., Hammel K.E. (2014). A Highly Diastereoselective Oxidant Contributes to Ligninolysis by the White Rot Basidiomycete Ceriporiopsis Subvermispora. Appl. Environ. Microbiol..

[52] Yelle D.J., Kaparaju P., Hunt C.G., Hirth K., Kim H., Ralph J., Felby C. (2013). Two-Dimensional NMR Evidence for Cleavage of Lignin and Xylan Substituents in Wheat Straw Through Hydrothermal Pretreatment and Enzymatic Hydrolysis. Bioenerg. Res..

[53] Giachi G., Capretti C., Macchioni N., Pizzo B., Donato I.D. (2010). A Methodological Approach in the Evaluation of the Efficacy of Treatments for the Dimensional Stabilisation of Waterlogged Archaeological Wood. J. Cult. Herit..

[54] Grattan D.W., Pearson C. (1987). 3-Waterlogged Wood. Conservation of Marine Archaeological Objects.

[55] Broda M., Majka J., Olek W., Mazela B. (2018). Dimensional Stability and Hygroscopic Properties of Waterlogged Archaeological Wood Treated with Alkoxysilanes. Int. Biodeter. Biodegr..

[56] Broda M., Curling S.F., Spear M.J., Hill C.A. (2019). Effect of Methyltrimethoxysilane Impregnation on the Cell Wall Porosity and Water Vapour Sorption of Archaeological Waterlogged Oak. Wood Sci. Technol..

[57] Van Opdenbosch D., Dörrstein J., Klaithong S., Kornprobst T., Plank J., Hietala S., Zollfrank C. (2013). Chemistry and Water-Repelling Properties of Phenyl-Incorporating Wood Composites. Holzforschung.

[58] Hill C.A.S., Farahani M.R.M., Hale M.D.C. (2004). The Use of Organo Alkoxysilane Coupling Agents for Wood Preservation. Holzforschung.

[59] Pesti J., Larson G.L. (2016). Tetramethyldisiloxane: A Practical Organosilane Reducing Agent. Org. Process Res. Dev..

[60] Larson G.L., Fry J.L. (2008). Ionic and Organometallic-Catalyzed Organosilane Reductions. Organic Reactions.

[61] Marciniec B. (2009). Hydrosilylation: A Comprehensive Review on Recent Advances. Advances in Silicon Science.

[62] Hossain M.A., Rahaman M.S., Yelle D., Shang H., Sun Z., Renneckar S., Dong J., Tulaphol S., Sathitsuksanoh N. (2021). Effects of Polyol-Based Deep Eutectic Solvents on the Efficiency of Rice Straw Enzymatic Hydrolysis. Ind. Crops. Prod..

[63] Yelle D.J., Ralph J., Frihart C.R. (2008). Characterization of Nonderivatized Plant Cell Walls Using High-Resolution Solution-State NMR Spectroscopy. Magn. Reson. Chem..

[64] Methyltrimethoxysilane(1185-55-3) 13C NMR. https://www.chemicalbook.com/SpectrumEN_1185-55-3_13CNMR.htm.

[65] Safaei-Ghomi J., Nazemzadeh S.H. (2017). Ionic Liquid-Attached Colloidal Silica Nanoparticles as a New Class of Silica Nanoparticles for the Preparation of Propargylamines. Catal. Lett..

[66] 1-(Aminoformylmethyl)Pyridinium Chloride(41220-29-5) 13C NMR. https://www.chemicalbook.com/SpectrumEN_41220-29-5_13CNMR.htm.

[67] Wieszczycka K., Filipowiak K., Wojciechowska I., Buchwald T., Siwińska-Ciesielczyk K., Strzemiecka B., Jesionowski T., Voelkel A. (2021). Novel Highly Efficient Ionic Liquid-Functionalized Silica for Toxic Metals Removal. Sep. Purif. Technol..

[68] Scott A.F., Gray-Munro J.E., Shepherd J.L. (2010). Influence of Coating Bath Chemistry on the Deposition of 3-Mercaptopropyl Trimethoxysilane Films Deposited on Magnesium Alloy. J. Colloid Interface Sci..

[69] The NMR of (3-Mercaptopropyl)Trimethoxysilane. http://www.hanhonggroup.com/nmr/nmr_en/MR040137.html.

[70] Wipfelder E., Höhn K. (1994). Epoxysiloxane Resins by the Condensation of 3-Glycidyloxypropyltrimethoxysilane with Diphenylsilanediol. Angew. Makromolek. Chem..

[71] 3-Glycidoxypropyltrimethoxysilane(2530-83-8) 13C NMR. https://www.chemicalbook.com/SpectrumEN_2530-83-8_13CNMR.htm.

[72] Li S., Kong X., Feng S. (2015). Preparation of Uniform Poly(Urea–Siloxane) Microspheres through Precipitation Polymerization. RSC Adv..

[73] Karasiewicz J., Krawczyk J. (2020). Thermodynamic Analysis of Trisiloxane Surfactant Adsorption and Aggregation Processes. Molecules.

