# Supplementary material for: Reactivity of Waterlogged Archeological Elm Wood with Organosilicon Compounds Applied as Wood Consolidants: 2D 1H–13C Solution-State NMR Studies
Source: Molecules. 2022 May 25;27(11):3407. doi: 10.3390/molecules27113407 (PMC9181845; doi:10.3390/molecules27113407)
Supplement: Supplementary file 1 [file molecules-27-03407-s001.zip › molecules-1728427-supplementary.pdf]

*Supplementary Information*

# Reactivity of Waterlogged Archeological Elm Wood with Organosilicon Compounds Applied as Wood Consolidants: 2D $^{1}H$–$^{13}C$ Solution-State NMR Studies

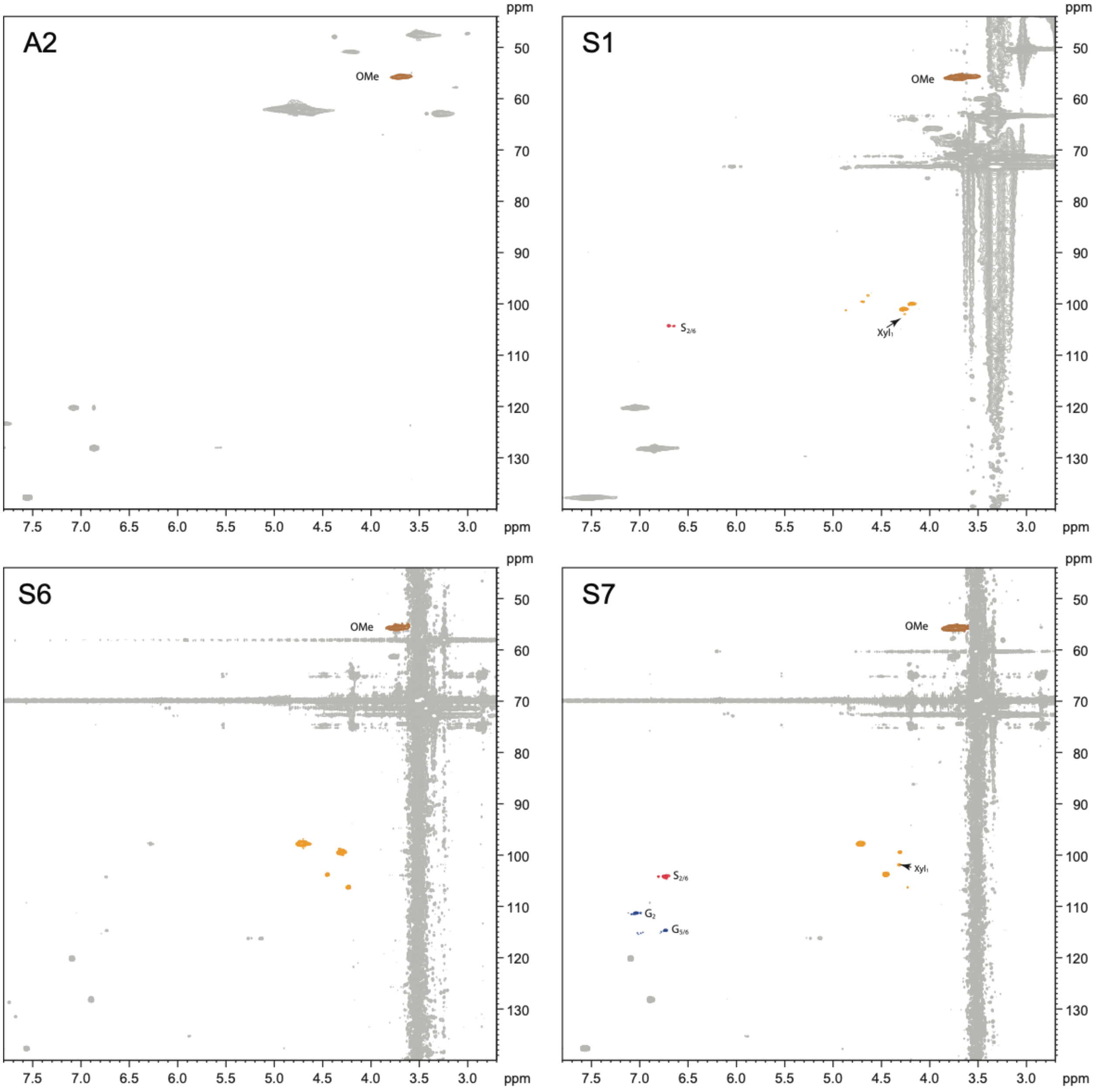


**Figure S1.** Partial 2D NMR spectra of organosilicon-treated wood showing 1-[3-(trimethoxysilyl)propyl]pyridinium chloride (A2), 1,3-bis(3-glycidyloxypropyl)-1,1,3,3-tetramethyldisiloxane (**S1**), 1,3,5,7-tetrakis(3-polyethoxypropyl)-1,3,5,7-tetramethyltetracyclosiloxane methoxy terminated (**S6**), 3-[3-(hydroxy)(polyethoxypropyl)]1,1,1,3,5,5,5-heptamethyltrisiloxane (**S7**). The colored contours and labels correspond to the chemical structures shown in Figure 3.

**Table S1.** Chemical shifts of functional groups present in selected organosilicon compounds used in this study.

| Organo-silicon applied | Functional group | $^{1}$H δ (ppm) | $^{13}$C δ (ppm) | Reference |
|---|---|---|---|---|
| A1 | methoxyl | 3.558 | 45 | [1] |
| | methyl | 0.124 | -10 | |
| | methoxyl | 3.558 | 45 | [1,2] |
| A2 | A, B, C/2, 3, 4 (pyridinium chloride ring) | 8.847, 8.671, 8.157 | 147,61, 146.59, 128.90 | [3] (**$^{1}$H NMR ($CDCl_3$) δ in ppm**: 8.90 (s, 1H, OH); 8.60 (d, 1H, $H_{py2}$); 8.05 (d, 1H, $H_{py6}$); 7.95 (d, 1H, $H_{py4}$); 7.30 (t, 1H, $H_{py5}$); 3.96 (t, $CH_2$-Cl); 3.57 (s, 9H, $CH_3$) 3.00 (t, 2H, $CH_2$); 2.20 (q, 2H, $CH_2$); 1.65 (t, 2H, $CH_2$); 1.55 (t, 2H, $CH_2$); 1.4-1.15 (m, 16H, $CH_2$); 0.87 (t, 3H, $CH_3$), 0.65 (t, 2H, $CH_2$) [4] **$^{13}$C NMR ($CDCl_3$) δ in ppm**: 156.5 (oxime C=N),147.5 ($C_{py2}$),144.6 ($C_{py6}$) 137.7($C_{py\,4}$); 134.3 ($C_{py5}$) 123.9 ($C_{py3}$) 52.7 ($CH_3$-O); 48.6 ($CH_2$-$N^+$), 41.8 $CH_2$-C=O), 32.3 ($CH_2$), 31.2 ($CH_2$), 29.6 ($CH_2$), 29.5 ($CH_2$), 29.4 ($CH_2$), 29.3 ($CH_2$), 24.5 ($CH_2$), 23.6 ($CH_2$), 18.5 ($CH_2$), 13.8 ($CH_3$); 10.7 ($CH_2$), 7.9 ($CH_2$-Si) [4] |
| A3 | 3B, 2C, (D), 4E | 2.537, 1.72, (1.356) HS, 0.76 | 27.54 (2,3), 8.27 | [5,6] |
| | methoxyl | 3.569 | 50.52 | |
| A4 | methoxyl | 3.558 | 45 | [1] |
| S1 | 1, 2, 3, 4, 5, 6, 7 | na | 73.58, 71.46, 50.89, 50.51, 44.27, 22.89, 5.32 | [7,8] |
| S2 | –Si–CH3, –Si–CH2–, –Si–CH2–CH2–, H | 0.06, 0.52, 1.44, 0 | 0.00, 14.73, 27.02 - C4 (44.72) | [9] |
| | -$NH_2$ | 1.14 | na | |
| S6 | O-Si(CH3)-O, Si-CH2, Si-CH2-CH2, Si-CH2-CH2-CH2, Si-CH2-CH2-CH2-O-CH2/_CH2 | -0.1, 0.4, 1.5, 3.4, 3.55 | 3, -1, 14, 24, 62, 70 | [10] |
| | OH | 3.55 | na | |
| S7 | -Si-CH3, O-Si(CH3)-O, Si-CH2, Si-CH2- | 0, -0.1, 0.4, 1.5, 3.4, 3.55 | 3, -1, 14, 24, 62, 70 | [10] |

| | |
|---|---|
| CH2, Si-CH2-<br>CH2-CH2, Si-<br>CH2-CH2-CH2-<br>O-CH2/_CH2 | |

na = not available.

**References:**


64. Methyltrimethoxysilane(1185-55-3) 13C NMR Available online: https://www.chemicalbook.com/SpectrumEN_1185-55-3_13CNMR.htm (accessed on 29 April 2022).
65. Safaei-Ghomi, J.; Nazemzadeh, S.H. Ionic Liquid-Attached Colloidal Silica Nanoparticles as a New Class of Silica Nanoparticles for the Preparation of Propargylamines. *Catal. Lett.* **2017**, *147*, 1696–1703, doi:10.1007/s10562-017-2079-4.
66. 1-(AMINOFORMYLMETHYL)PYRIDINIUM CHLORIDE(41220-29-5) 13C NMR Available online: https://www.chemicalbook.com/SpectrumEN_41220-29-5_13CNMR.htm (accessed on 29 April 2022).
67. Wieszczycka, K.; Filipowiak, K.; Wojciechowska, I.; Buchwald, T.; Siwińska-Ciesielczyk, K.; Strzemiecka, B.; Jesionowski, T.; Voelkel, A. Novel Highly Efficient Ionic Liquid-Functionalized Silica for Toxic Metals Removal. *Sep. Purif. Technol.* **2021**, *265*, 118483, doi:10.1016/j.seppur.2021.118483.
68. Scott, A.F.; Gray-Munro, J.E.; Shepherd, J.L. Influence of Coating Bath Chemistry on the Deposition of 3-Mercaptopropyl Trimethoxysilane Films Deposited on Magnesium Alloy. *J. Colloid Interface Sci.* **2010**, *343*, 474–483, doi:10.1016/j.jcis.2009.11.062.
69. The NMR of (3-Mercaptopropyl)Trimethoxysilane Available online: http://www.hanhong-group.com/nmr/nmr_en/MR040137.html (accessed on 29 April 2022).
70. Wipfelder, E.; Höhn, K. Epoxysiloxane Resins by the Condensation of 3-Glycidyloxypropyltrimethoxysilane with Diphenylsilanediol. *Angew. Makromolek. Chem.* **1994**, *218*, 111–126, doi:10.1002/apmc.1994.052180108.
71. 3-Glycidoxypropyltrimethoxysilane(2530-83-8) 13C NMR Available online: https://www.chemicalbook.com/SpectrumEN_2530-83-8_13CNMR.htm (accessed on 29 April 2022).
72. Li, S.; Kong, X.; Feng, S. Preparation of Uniform Poly(Urea–Siloxane) Microspheres through Precipitation Polymerization. *RSC Adv.* **2015**, *5*, 90313–90320, doi:10.1039/C5RA18140B.
73. Karasiewicz, J.; Krawczyk, J. Thermodynamic Analysis of Trisiloxane Surfactant Adsorption and Aggregation Processes. *Molecules* **2020**, *25*, 5669, doi:10.3390/molecules25235669.
